# Supplementary material for: Peering through the PSMA PET Lens: The Role of the European Association of Urology Biochemical Recurrence Risk Groups after Radical Prostatectomy
Source: Cancers (Basel). 2023 May 26;15(11):2926. doi: 10.3390/cancers15112926 (PMC10252094; doi:10.3390/cancers15112926)
Supplement: Supplementary file 1 [file cancers-15-02926-s001.zip › cancers-2406103-supplementary.pdf]

Supplementary Data

Cutoff values for kinetic PSA values

The area under the receiver operating characteristic (ROC) curve was 0.659 (95% CI 0.6-0.7; $p < 0.0001$) for the PSA value at the time of PET/CT, 0.622 (95% CI 0.6-0.7; $p = 0.049$) for PSAdt, and 0.693 (95% CI 0.6-0.7; $p < 0.0001$) for PSAvel.

The optimal cutoff values to differentiate a positive or negative PET-PSMA result were 0.5 ng/mL for the PSA value at the time of PET/CT, 4 months for PSAdt, and 0.4 ng/mL/year for PSAvel.

The 68Ga-PSMA-11 PET/CT was positive in 36.2% of patients who had a PSA value < 0.5 ng/mL at the time of PET/CT compared to 60.8% in patients who had a PSA value ≥ 0.5 ng/mL at the time of PET/CT ($p < 0.001$).

The 68Ga-PSMA-11 PET/CT was positive in 49.8% of patients who had a PSAdt > 4 months, compared to 66.4% in patients with a PSAdt ≤ 4 months ($p = 0.002$).

The 68Ga-PSMA-11 PET/CT was positive in 37.6% of patients with a PSAvel < 0.4 ng/mL/year, compared to 61% in patients with a PSAvel ≥ 0.19 ng/mL/year ($p < 0.001$).
